# The Effects of Sun Exposure and Pigmentation Phenotype on Prognosis in Metastatic Melanoma

**DOI:** 10.2340/actadv.v106.adv-2026-0388

**Published:** 2026-05-12

**Authors:** Teresa Svensson, Sebastian Sahlberg, Ana Carneiro, Karolin Isaksson, Kari Nielsen, Henrik Ekedahl

**Affiliations:** 1 Department of Clinical Sciences Lund, Lund University Cancer Center, Lund University, Lund, Sweden; 2 Department of Oncology, Skåne University Hospital Lund, Lund, Sweden; 3 Department of Surgery, Skåne University Hospital Kristianstad, Kristianstad, Sweden; 4 Department of Dermatology, Skåne University Hospital Lund, Lund, Sweden; 5 Department of Dermatology, Skåne University Hospital Helsingborg, Helsingborg, Sweden

**Keywords:** immune checkpoint inhibitors, melanoma, phenotype, ultraviolet rays

## Abstract

Ultraviolet radiation exposure and a fair pigmentation phenotype are well-established risk factors for primary cutaneous melanoma. However, the prognostic relevance of these risk factors is largely unexplored in metastatic disease. The aim of this study was to examine whether phenotypic characteristics and sun exposure patterns affect the prognosis of metastatic melanoma with immunotherapy available. In this retrospective study, 210 patients with stage IV melanoma were included. All patients were asked to answer a standardized questionnaire regarding risk factors for melanoma, including items on sun exposure habits and self-assessment of phenotypic features. Survival analyses were performed with overall survival as the endpoint. Patients who reported >5 severe sunburns during childhood had a significantly decreased risk of death compared to patients with 0–1 sunburn (HR 0.46, CI 0.25–0.85, *p*=0.013). A fair or intermediate pigmentation phenotype was associated with a decreased risk of death compared to a dark phenotype after adjustment for distant metastasis category (HR 0.57, CI 0.34–0.93, *p*=0.026). This study indicates that the number of severe sunburns during childhood is associated with a more favourable prognosis in metastatic melanoma. However, due to the limited size of the study population, further research is required to confirm these results.

**SIGNIFICANCE**

This study highlights potential prognostic factors in metastatic melanoma, namely the number of severe sunburns during childhood and pigmentation phenotype. These results may also have implications for the interpretation of results from studies including patients with different proportions of pigmentation phenotypes and sun exposure histories.

Cutaneous melanoma is an aggressive neoplasm of the skin with increasing incidence in western populations over the last decades (1). Several risk factors for melanoma are well-established. The major environmental risk factor is ultraviolet radiation (UVR) exposure, in particular a history of intermittent sun exposure and sunburn episodes (2). UVR causes direct damage to the DNA-strands, oxidative stress and inflammatory responses (3). An inherited risk factor is pigmentation phenotype. A higher ratio between the pigments pheomelanin and eumelanin causes a fairer pigmentation phenotype and less protection from UVR, leading to an increased risk of mutations. Consequently, red and blonde hair, freckles, fair skin and light eye colour are all risk factors for development of melanoma (4).

The prognosis of metastatic melanoma has historically been poor, but the introduction of immunotherapy in the last decade has markedly improved survival rates. Most frequently utilized types of immunotherapies are the immune checkpoint inhibitors (ICI), cytotoxic T-lymphocyte-associated antigen 4 (CTLA-4) inhibitors and programmed cell death 1 (PD-1) inhibitors (5). However, not all patients respond to ICI and available predictive markers, such as PD-L1, tumour mutational burden (TMB) and gene expression immune signatures, are insufficient for precise prediction of individual responses (5, 6).

While several risk factors for primary melanoma are well-documented, the prognostic relevance of these risk factors in metastatic disease in the immunotherapy era is uncertain. Previous studies of the prognostic value have mainly focused on genetic determinants of pigmentation, i.e., presence of melanocortin-1-receptor gene (MC1R) variants (7), and proxies for sun exposure, such as site of primary tumour (8–10). Interestingly, these studies indicate that MC1R variants associated with the red hair colour (RHC) phenotype and primary tumours on chronic sun exposed sites are associated with response to ICI. This suggests that phenotype and previous sun habits could offer prognostic value, which furthermore could have implications for how to interpret results from studies including patients with different proportions of pigmentation phenotypes and sun habits. Therefore, the aim of this study was to examine whether phenotypic characteristics and sun exposure patterns affect the prognosis in metastatic melanoma. We hypothesised that phenotypes and previous sun habits associated with a higher melanoma risk would be associated with a more favourable prognosis in stage IV melanoma.

## MATERIALS AND METHODS

This retrospective study cohort comprised melanoma patients from a Swedish population, identified in the BioMEL database (ClinicalTrials.gov (NCT05446155) (11). In Region Skåne, with approximately 1.5 million inhabitants, 806 new cases of cutaneous melanoma were registered during 2023, the majority being thin primary melanomas (12). Inclusion criteria in the current study were: a confirmed diagnosis of stage IV nonacral cutaneous melanoma, inclusion in the BioMEL database between 2013 and 2023 and having completed a patient questionnaire, although responding to all individual items was not required. Patients could have been enrolled in BioMEL at an earlier stage but were included in this study only if they developed stage IV disease. A total of 450 patients were identified in the database. Patients were excluded due to missing patient questionnaire (*n*=99), diagnosis of other melanoma subtypes (*n*=46) and lack of stage IV diagnosis (*n*=95). Consequently, 210 patients met the inclusion criteria (baseline characteristics are presented in Table I). Subtypes, including mucosal and acral melanomas, were excluded due to weaker association with sun exposure (arising in sun-shielded locations) combined with known poorer prognosis and lower response to immunotherapy (13). Most patients (*n*=133, 63%) initiated first-line treatment or were diagnosed (patients not receiving any systemic therapy) after August 2015, when PD-1 inhibitors were introduced as standard treatment at the Department of Oncology, Skåne University Hospital, Lund, Sweden. Clinical data on disease characteristics, treatment and follow-up were obtained from medical records and the cut-off date for follow-up and censoring was January 31, 2025. Staging was performed according to the 8^th^ AJCC staging edition. The reporting of this study was guided by the STROBE (Strengthening the Reporting of Observational Studies in Epidemiology) checklist (14).

**Table I.** Baseline characteristics of the study population, *n*=210

Gender, *n* (%)	
Male	133 (63)
Female	77 (37)
Age stage IV diagnosis, years, mean (SD)	66 (13)
Site primary, *n* (%)	
Head/Neck	29 (14)
Trunk	84 (40)
Upper extremity	16 (8)
Lower extremity	40 (19)
Unknown primary	29 (14)
Data missing	12 (6)
BRAF mutation, *n* (%)	
V600E	73 (35)
V600K	19 (9)
V600R	2 (1)
Wildtype	107 (51)
Data missing	9 (4)
Histopathological subtype of primary melanoma, *n* (%)	
Superficial spreading melanoma	70 (33)
Nodular melanoma	61 (29)
Lentigo maligna melanoma	9 (4)
Other skin melanoma	4 (2)
Unknown primary	29 (14)
Data missing	37 (18)
Breslow, mm, median (IQR)	3.1 (1.3–5.0)
Previous adjuvant treatment, *n* (%)	
ICI or BRAF inhibitor+MEK inhibitor	22 (10)
No previous treatment	188 (90)
Treatment stage IV first-line, *n* (%)	
PD-1 inhibitor+CTLA-4 inhibitor	34 (16)
PD-1 inhibitor	60 (29)
CTLA-4 inhibitor	22 (10)
BRAF inhibitor+MEK inhibitor	41 (20)
Chemotherapy	28 (13)
Other treatment	6 (3)
No treatment	19 (9)
Treatment stage IV second-line, *n* (%)	
PD-1 inhibitor+CTLA-4 inhibitor	22 (10)
PD-1 inhibitor	23 (11)
CTLA-4 inhibitor	18 (9)
BRAF inhibitor+MEK inhibitor	26 (12)
Chemotherapy	10 (5)
Other treatment	5 (2)
No second-line treatment	106 (50)
Treatment stage IV third-line, *n* (%)	
PD-1 inhibitor+CTLA-4 inhibitor	6 (3)
PD-1 inhibitor	12 (6)
CTLA-4 inhibitor	3 (1)
BRAF inhibitor+MEK inhibitor	11 (5)
Chemotherapy	12 (6)
Other treatment	2 (1)
No third-line treatment	164 (78)
Distant metastasis (M) category^a^, *n* (%)	
M1a	34 (16)
M1b	52 (25)
M1c	71 (34)
M1d	53 (25)
Lactate dehydrogenase^b^, *n* (%)	
Elevated	54 (26)
Not elevated	70 (33)
Data missing	86 (41)

^a^Indicates site of distant metastasis according to AJCC 8^th^ edition. ^b^Measured within±2 weeks of stage IV diagnosis or, if unavailable, within 2 weeks before first-line treatment; values unavailable in both intervals classified as unknown; elevation defined according to institutional upper reference limit.

ICI: immune checkpoint inhibitor; IQR: interquartile range; SD: standard deviation.

Upon inclusion in BioMEL, patients were asked to fill out a previously validated questionnaire (15). The questionnaire was administered by a research nurse, and each patient completed it on a single occasion. The questionnaire consisted of defined questions regarding risk factors for primary melanoma, including items on sun habits and phenotypic features. Definitions of categorical variables are presented in Table SI. For the current study, a phenotypic index was constructed based on the relative risk of primary melanoma, as reported in a meta-analysis (4). Phenotypic characteristics included in the index were presence of freckles, ability to achieve a tan, eye colour and hair colour. Weighted points were assigned for each phenotypic feature according to its associated risk, while absence of the feature received no points (Table SII). The points for each patient were summed, and patients were classified into dark, intermediate and fair phenotypes according to the total score (Table SIII).

### Statistical analysis

Survival analyses were performed with overall survival (OS) as primary endpoint, defined as time from date of stage IV melanoma diagnosis to death from any cause, with censoring at the last follow-up date for survivors. Survival curves were generated by the Kaplan–Meier method and *p*-values were calculated using the log-rank test. Cox regression analysis for univariate and multivariate analyses was performed, and the results are presented as hazard ratios (HR) and 95 % confidence intervals (CI). The *p*-values were 2-sided and a *p*-value less than 0.05 was considered statistically significant. For comparisons between characteristics in different groups, χ^2^, ANOVA and Mann–Whitney test were performed. Results for metric variables with a normal distribution are presented as means with standard deviations (SD), whereas results for metric variables with a skewed distribution are presented as medians with interquartile ranges (IQR). Patients with missing data were excluded from analyses requiring those variables. All statistical analyses were performed in IBM SPSS Statistics (version 29.0, Armonk, NY: IBM Corp).

## RESULTS

**Fig. 1.**
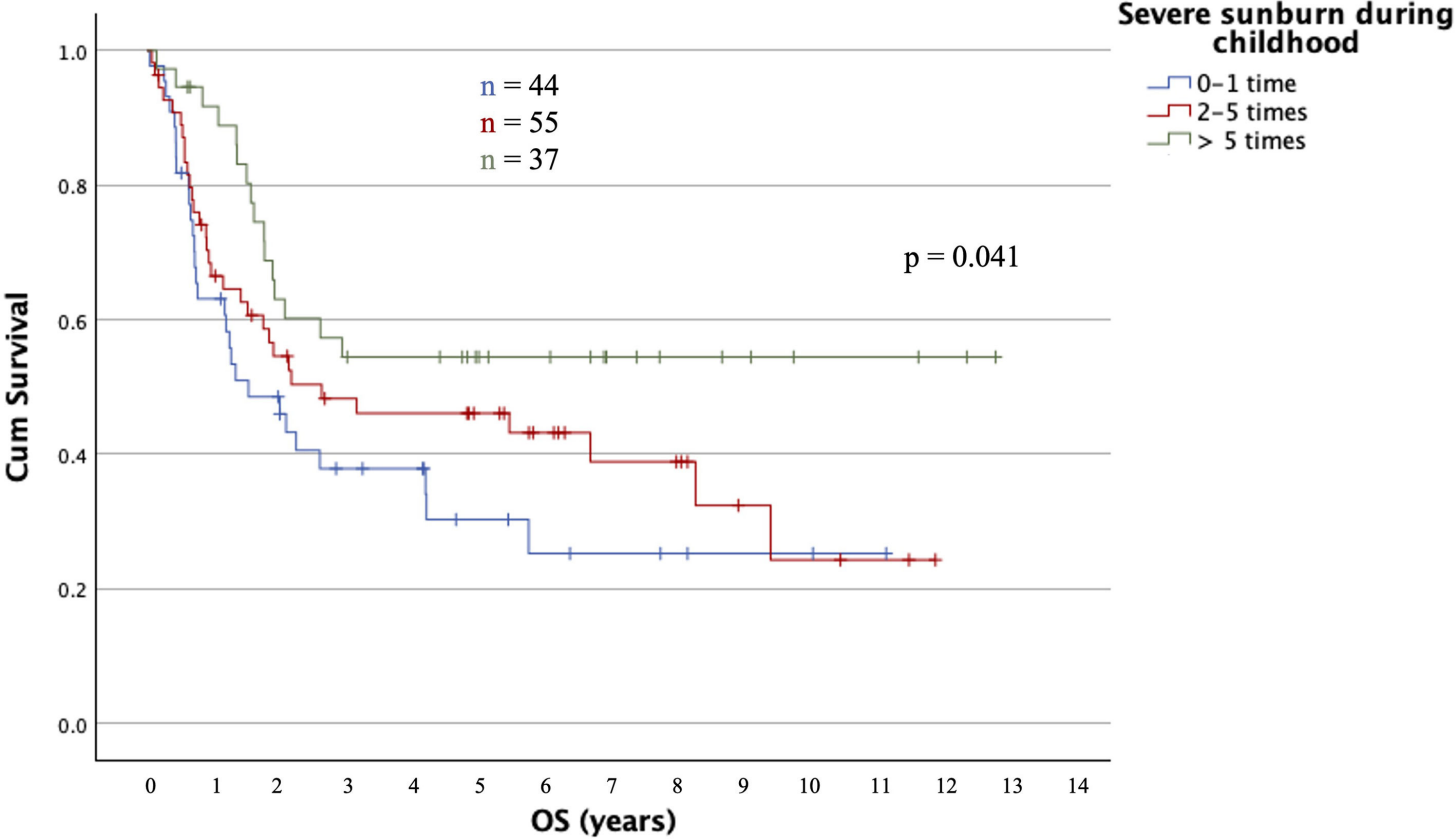
Kaplan–Meier curves showing overall survival (OS) for groups based on number of severe sunburns during childhood. Log-rank test was performed, and the result is presented as a *p*-value.

Univariate analyses of the associations between patients' risk factors for primary melanoma and OS in stage IV disease are presented in Table II. An increasing number of reported severe sunburns during childhood was significantly associated with a longer OS (*p*=0.046). Median OS for patients with >5 severe sunburns was not reached, while for patients with 2–5 and 0–1 severe sunburns median OS was 2.6 and 1.5 years, respectively (Fig. 1). Patients with a history of >5 severe sunburns during childhood had a significantly decreased risk of death compared to patients with 0–1 severe sunburn (HR 0.46, CI 0.25–0.85, *p*=0.013), but the difference between patients who reported 2–5 and 0–1 severe sunburns during childhood was not statistically significant (HR 0.76, CI 0.46–1.26, *p*=0.282). When adjusting for AJCC distant metastasis (M) category, the results were consistent (Table II).

**Table II.** Cox regression of overall survival according to phenotype characteristics, sun exposure and sunburn history

		Unadjusted			Adjusted for AJCC M category		
	n^a^	HR	95 % CI	*p*	HR	95 % CI	*p*
Freckles							
No	152	Ref			Ref		
Yes	45	1.07	0.70–1.62	0.760	1.17	0.77–1.78	0.456
Hair colour							
Black/brown	40	Ref		0.345	Ref		0.449
Blond	150	1.07	0.67–1.69	0.786	0.99	0.62–1.56	0.950
Red	15	0.58	0.23–1.42	0.229	0.58	0.23–1.43	0.237
Eye colour							
Dark brown	14	Ref			Ref		
Blue/green/hazel	191	0.67	0.35–1.27	0.218	0.78	0.41–1.51	0.461
Tanning ability							
Moderate/dark	77	Ref			Ref		
No tan/light	126	0.93	0.64–1.35	0.689	0.82	0.56–1.20	0.306
Phenotype index							
Dark phenotype	28	Ref		0.312	Ref		0.072
Intermediate phenotype	129	0.69	0.42–1.15	0.154	0.55	**0.33–0.91**	**0.022**
Fair phenotype	34	0.65	0.35–1.22	0.182	0.60	0.32–1.14	0.157
Times sunbathing each year							
Never	88						
1–14 times	85	Ref			Ref		
15–30 times	21	1.09^b^	0.66–1.77^b^	0.744^b^	1.28^b^	0.78–2.11^b^	0.336^b^
>30 times	9						
Outdoor occupation							
<6 h each week	159	Ref			Ref		
≥6 h each week	48	0.75	0.48–1.17	0.204	0.71	0.46–1.12	0.142
Indoor tanning exposure							
No	126	Ref			Ref		
Yes	81	1.07	0.75–1.54	0.700	1.11	0.77–1.60	0.583
Severe sunburn during childhood							
0–1 time	44	Ref		**0.046**	Ref		**0.034**
2–5 times	55	0.76	0.46–1.26	0.282	0.80	0.49–1.33	0.399
>5 times	37	0.46	**0.25–0.85**	**0.013**	0.44	**0.23–0.82**	**0.010**
Sun holidays							
<Once per year	163	Ref					
≥Once per year	39	1.16	0.74–1.82	0.521	1.00	0.63–1.58	0.997

^a^Cases with missing data were excluded from tabulations and analyses. ^b^< 15 times vs≥15 times.

AJCC: American Joint Committee on Cancer Staging Manual eighth edition; CI: confidence interval; HR: hazard ratio.

There was no significant difference between any of the individual phenotypic characteristics and OS (Table II). A phenotypic index based on eye, hair and skin colour and presence of freckles was used to classify patients into fair, intermediate and dark pigmentation phenotypes. Patients with the fair and intermediate phenotypes had numerically lower risks of death compared to patients with dark phenotype (HR 0.65, CI 0.35–1.22, *p*=0.182 and HR=0.69, CI 0.42–1.15, *p*=0.154, respectively, Table II). After adjustment for M category, the difference in OS between the intermediate and dark phenotype was significant (HR 0.55, CI 0.33–0.91, *p*=0.022), although the overall difference between the three groups was not significant (*p*=0.072). When patients with the dark phenotype were compared to the combined group of patients with intermediate and fair phenotypes, there was a nonsignificant trend towards a decreased risk of death for the intermediate/fair phenotypes (HR 0.68, CI 0.42–1.12, *p*=0.131), but when adjusting for M category, the difference was significant (HR 0.57, CI 0.34–0.93, *p*=0.026).

The number of times sunbathing each year, indoor tanning exposure, outdoor occupation and number of sun holidays were not associated with OS (Table II). Common prognostic factors, namely age, gender, baseline M category and lactate dehydrogenase (LDH), were also assessed in relation to OS. Older age at stage IV diagnosis, higher baseline M category and LDH>upper limit of normal were significantly associated with an increased risk of death, while gender was not (data not shown).

To assess whether the observed survival advantage for patients with >5 severe sunburns during childhood could be explained by other factors, the groups with varying numbers of severe childhood sunburns were compared regarding other risk factors, clinical characteristics, prognostic factors in stage IV disease and level of education. Number of severe sunburns during childhood was not associated with any of these factors (Table SIV). However, there was a nonsignificant trend towards a higher proportion of patients with freckles among patients with >5 severe childhood sunburns compared to 2–5 and 0–1 (35% vs. 25 % vs. 18%, respectively, *p*=0.146).

Previous studies have used head/neck location of the primary melanoma as a surrogate for chronic sun exposure (8–10). In this study, we found anticipated associations between primary tumours on extremities and female sex as well as between head/neck location and BRAF V600K mutations (Table III). However, primary site was not associated with sun habits or outdoor work, but patients with head/neck melanoma had more often dark hair and a higher frequency of a dark phenotype index compared to patients with melanomas on trunk and extremities (Table III). To test the independent prognostic impact of the phenotype index and tumour site, we performed a multivariate Cox regression analysis. Here, a dark phenotype and higher M category were significantly associated with a poorer survival (HR 1.83, CI 1.04–3.22, *p*=0.037 and HR 2.55, CI 1.62–4.02, *p*=<0.001, respectively), but tumour site and sex were not (HR 1.49, CI 0.82–2.71, *p*=0.190 for trunk compared to head/neck, HR 1.22, CI 0.64–2.34, *p*=0.544 for extremity compared to head/neck, and HR 0.75, CI 0.48–1.2, *p*=0.184 for male compared to female).

**Table III.** Characteristics of primary location of melanoma (*n*=169)

	Head/neck (*n*=29)	Trunk (*n*=84)	Extremity (*n*=56)	*p*
Gender, *n* (%)				**0.043^b^**
Male	20 (69)	57 (68)	27 (48)	
Female	9 (31)	27 (32)	29 (52)	
Age stage IV (years, mean (SD)	65 (13)	67 (13)	65 (13)	0.501^c^
Distant metastasis (M) category^a^, *n* (%)				0.713^d^
M1a	4 (14)	9 (11)	13 (23)	
M1b	7 (24)	23 (27)	12 (21)	
M1c	10 (34)	30 (36)	22 (39)	
M1d	8 (28)	22 (26)	9 (16)	
BRAF mutation, *n* (%)				**0.016^e^**
V600E	8 (29)	28 (33)	26 (46)	
V600K	6 (21)	10 (12)	0	
Wildtype	14 (50)	43 (51)	26 (46)	
Unknown	0	3 (4)	4 (7)	
Breslow (mm), median (IQR)	4.1 (2.0–6.9)	3.5 (1.2–5.0)	2.5 (1.5–4.8)	0.160^f^
Treatment stage IV, *n* (%)				0.528^b^
ICI	22 (76)	59 (70)	44 (79)	
No ICI	7 (24)	25 (30)	12 (21)	
Hair colour, *n* (%)				**0.038^g^**
Black/brown	10 (34)	11 (13)	10 (18)	
Blond	19 (66)	69 (82)	40 (71)	
Red	0	4 (5)	4 (7)	
Unanswered	0	0	2 (4)	
Phenotype index, *n* (%)				**0.031^h^**
Dark	8 (28)	8 (10)	4 (7)	
Intermediate/fair	21 (72)	69 (82)	45 (80)	
Unanswered	0	7 (8)	7 (13)	
Times sunbathing each year, *n* (%)				0.252^i^
<15 times	26 (90)	67 (80)	48 (86)	
≥15 times	2 (7)	16 (19)	7 (13)	
Unanswered	1 (3)	1 (1)	1 (2)	
Outdoor occupation, *n* (%)				0.754^b^
<6 h each week	22 (76)	63 (75)	45 (80)	
≥6 h each week	7 (24)	21 (25)	11 (20)	
Severe sunburn during childhood, *n* (%)				0.876^j^
0–1 time	9 (31)	16 (19)	13 (23)	
2–5 times	7 (24)	23 (27)	15 (27)	
>5 times	5 (17)	15 (18)	10 (18)	
Unanswered	8 (28)	30 (36)	18 (32)	

^a^Indicates site of distant metastasis according to AJCC 8^th^ edition. ^b^χ^2^ test. ^c^Anova. ^d^M1a+M1b vs M1c+M1d, χ^2^ test. ^e^BRAF V600E mutation vs V600K mutation vs wildtype, χ^2^ test. ^f^Kruskal wallis test. ^g^Black/brown vs blond+red, χ^2^ test. ^h^Dark phenotype vs intermediate+fair phenotype, χ^2^ test. ^i^<15 times vs ≥15 times, χ^2^ test. ^j^0–1 time vs 2–5 times vs >5 times, χ^2^ test. ICI: immune checkpoint inhibitor; IQR: interquartile range; SD: standard deviation.

## DISCUSSION

Sun exposure and pigmentation phenotype are well known risk factors associated with melanoma incidence (3, 4). How these clinical risk factors affect survival in melanoma is less established, especially after immunotherapy was introduced. In this study, an increasing number of severe sunburns during childhood was significantly associated with an improved OS in stage IV melanoma. This might be explained by a previously described association between a history of blistering sunburns and a higher TMB (16). It has been shown that TMB, which is defined as the number of genetic alterations in a tumour, correlates with the number of neoantigens present, which in turn correlates with response to ICI (17, 18).

The relationship between pigmentation phenotype and sun exposure is complex. Prior studies have indicated that individuals with the RHC phenotype are more likely to practise sun avoidance (19, 20). Thus, individuals with the RHC phenotype would be expected to have lower cumulative sun exposure. This inverse relationship between a fairer phenotype and sun exposure could thus diminish the likelihood of detecting a correlation between each of the risk factors and prognosis. At the same time, it has been shown that fair skin type, light eye colour and lighter hair were associated with more sunburns for children up to 7 years of age (21), indicating that severe sunburns are a result of the combination of the 2 factors: high intermittent sun exposure and a fair pigmentation phenotype (enabling severe sunburns).

Interestingly, melanocortin-1-receptor gene (MC1R) variants, associated with the RHC phenotype (MC1R-R alleles), have also been associated with high TMB (5, 22). In line with these findings, a recent study indicated that individuals with MC1R-R alleles have a better response to ICI (7). However, no significant survival benefits were demonstrated regarding the phenotypic features skin and hair colour, whereas sun habits were not analysed (7). This is consistent with our results, since no significant effect on prognosis was seen based on separate phenotype characteristics. Yet, a dark phenotype, defined by an index of combined phenotypic risk factors, was in our study significantly associated with a poorer OS. That MC1R-R alleles have been associated with a more favourable prognosis, while phenotypic features were not as clearly associated, could be attributed to MC1R-R alleles resulting in higher TMB partly independent of pigmentation. There are indications supporting this; in a previous study, all classes of mutations were increased in patients with MC1R-R alleles, not only the ones typically associated with UVR (22). Furthermore, studies have shown that some MC1R variants are risk factors for melanoma due to nonpigmentation pathways (23, 24). Another explanation might be a rather homogenous phenotype in the cohort, i.e., a small number of patients had dark eye colour and a hair colour other than blond. Thus, the MC1R-genotype and the phenotype index might better discriminate the patients in prognostic groups given a limited sample size.

Regarding the effect of chronic sun exposure, none of the questions in the questionnaire in our study clearly captured the entire aspect of this isolated event. For example, times sunbathing each year at least partly includes intermittent exposure, and outdoor occupation does not include all activities resulting in chronic sun exposure. Previous studies have used location of the primary melanoma on head and neck as a proxy for cumulative sun exposure. Indeed, head/neck melanomas have been associated with higher TMB, which in turn was linked to more frequent responses to ICI treatment (8). Another study showed that head/neck melanomas had the most significant improvement in prognosis since ICI was introduced (9). Moreover, it has been shown that patients with head/neck melanomas had longer progression free survival and OS compared to other primary sites when treated with immunotherapy, particularly single PD-1 inhibitor (10). In our study, tumour site was not associated with prognosis. However, head/neck melanoma was associated with a dark phenotype, suggesting that individuals with a dark phenotype require a higher UVR exposure, as a result of high cumulative/chronic sun exposure, to develop melanoma. To analyse the independent associations of pigmentation phenotype and primary melanoma location, we performed a multivariate analysis. In this model, phenotype was significantly associated with OS, whereas the lower HRs for head/neck location compared to trunk and extremities were not significant. This suggests that pigmentation phenotype is an independent prognostic factor and the lack of significant association between location and OS might be due to the limited size of the study population.

It is well known that socioeconomic status (SES) affects health, and associations between SES and melanoma survival have previously been shown (25). A well-established and frequently used indicator of SES is level of education (26). Number of reported severe sunburns could possibly be influenced by SES, for example, individuals with a higher SES might afford trips to sunny locations resulting in sunburns. However, in our study, there was no association between level of education and the number of severe sunburns during childhood. Hence, no support for SES being a confounding factor was found. Although education is a frequent indicator of SES, it might not however fully capture SES.

### Limitations

This study is limited by its retrospective design, which implies an increased risk for confounders and selection bias, as patients with the worst prognosis may have been underrepresented (due to death before inclusion). Therefore, results from a retrospective study may not fully represent the real-world population. The utilization of the questionnaire allows patients’ interpretations of subjective questions regarding pigment, and it can be challenging for patients to accurately report sun exposure earlier in life. Although, the questionnaire used in the study is previously validated and covers a wide range of important intrinsic and extrinsic risk factors (15). The small study population is another limiting factor, restricting analyses of several subgroups, in particular the possibility to analyse the effect of different treatments. Furthermore, the results from this Swedish cohort are not applicable to populations outside of Northern Europe, where phenotypic characteristics and sun exposure habits may differ substantially. The generalizability is further limited by the exclusion of noncutaneous and acral melanomas. Consequently, the results are applicable solely to nonacral cutaneous melanomas and not the entire metastatic melanoma population.

### Conclusion

This study showed an association between a higher number of severe sunburns during childhood and longer overall survival in patients with metastatic melanoma, which to our knowledge has not been previously described in a modern setting where immunotherapy is available. Further research is needed to confirm the results, given the small study population, and to delineate the underlying mechanisms.
