# Supplementary material for: The Effects of Sun Exposure and Pigmentation Phenotype on Prognosis in Metastatic Melanoma
Source: Acta Derm Venereol. 2026 May 12;106:0388. doi: 10.2340/actadv.v106.adv-2026-0388 (PMC13168784; doi:10.2340/actadv.v106.adv-2026-0388)
Supplement: Supplementary file 1. [file ActaDv-106-0388-s0001.pdf]

Supplementary material has been published as submitted. It has not been copyedited, or typeset by Acta Dermato-Venereologica

SUPPLEMENTARY METHODS

Table SI: Definitions of categorical variables based on the patient questionnaire

Variable	Question wording	Original answer options
Freckles	Do you develop freckles during first sun exposure of the year or when using a tanning bed?	Yes, no
Hair colour	What was/is your natural hair colour between the ages of 15 and 25?	Black, brown, dark blond, medium blond, light blond, red[1]
Eye colour	What is your eye colour (if it has changed, choose the colour you had at age 18)?	Dark brown, blue, green, hazel[2]
Tanning ability	How does your skin typically respond after several consecutive days of sun exposure?	Deep tan, moderate tan, light tan, no tan[3]
Times sunbathing each year	How often do you actively sunbathe outdoors (with the intention of tanning) during the summer months (April–September)?	Never, 1–14 times, 15–30 times, >30 times
Outdoor occupation	What proportion of your working time do you spend outdoors?	≤5 hours per week, 6-20 hours per week, > 20 hours per week[4]
Indoor tanning exposure	Have you ever used a tanning bed?	Yes, no
Severe sunburns during childhood	Have you ever experienced severe sunburn with pronounced redness and pain in the skin during childhood (ages 0–15)?	0-1 time, 2-5 times, >5 times
Sun holidays	Do you usually travel abroad for sunbathing and swimming?	Never, occasionally, once per year, two or more times per year[5]
Level of education	Do you have any education above compulsory school level?	No, secondary school, upper secondary school, university, folk high school, vocational school[6]

For analyses, similar response categories were merged to form larger groups. Footnotes below show merged groups.
[1] Black/brown, blond, red.
[2] Dark brown, blue/green/hazel
[3] Deep/moderate, light/no tan
[4] < 6 hours each week, ≥6 hours each week
[5] < Once per year, ≥ once per year
[6] Upper secondary school or below, above upper secondary school

CREATION OF PHENOTYPIC INDEX

Table SII: Risk classification of phenotypic features

Phenotypic feature	n*	Relative risk	Points
Freckles			
Yes	45	2.10	1
No	155	Ref	0
Skin/tan			
No tan/light tan	127	2.06	1
Moderate/deep tan	79	Ref	0
Eye colour			
Blue/green/hazel/grey	194	1.47-1.61	1
Dark brown	14	Ref	0
Hair colour			
Red	15	3.64	2
Blond	152	1.96	1
Brunette/black	41	Ref	0

Calculation of index according to relative risks from a meta-analysis (1). Presence of each phenotypic features associated with higher risk was given points, while an absence of the feature was not given any point.

* Cases with missing/unanswered data were excluded from grouping and subsequent analyses.

Table SIII: Distribution of patients into phenotype groups

Index points	n	Phenotype group
0	4	Dark
1	24	
2	55	Intermediate
3	77	
4	23	Fair
5	11	

The points shown in table S2 were summed for each of the patients, and the patients were divided into three different pigmentation phenotype groups based of the total score of points.

SUPPLEMENTARY RESULTS

Table SIV. Characteristics of sunburns during childhood groups (n = 136)

	0-1 time (n = 44) n (%)	2-5 times (n =55) n (%)	> 5 times (n = 37) n (%)	p
Gender				0.960[3]
Male	32 (73)	40 (73)	26 (70)	
Female	12 (27)	15 (27)	11 (30)	
Age stage IV (years)				0.374[4]
Mean (SD)	63 (13)	66 (15)	62 (11)	
Site primary				0.893[5]
Head/Neck	8 (18)	7 (13)	5 (14)	
Trunk	15 (34)	23 (42)	15 (41)	
Upper extremity	5 (11)	8 (15)	1 (3)	
Lower extremity	8 (18)	7 (13)	8 (22)	
Unknown primary	8 (18)	7 (13)	5 (14)	
Data missing	0	3 (5)	3 (8)	
Breslow (mm)				0.624[6]
Median (IQR)	3.5 (1.9-4.6)	3.0 (1.2-4.2)	3.0 (0.9-5.0)	
Distant metastasis (M) category[1]				0.528[7]
M1a	5 (11)	13 (24)	4 (11)	
M1b	11 (25)	11 (20)	14 (38)	
M1c	16 (36)	20 (36)	9 (24)	
M1d	12 (27)	11 (20)	10 (27)	
BRAF mutation				0.756[8]
V600E	15 (34)	22 (40)	13 (35)	
V600K	3 (7)	6 (11)	5 (14)	
V600R	1 (2)	0	1 (3)	
Wildtype	24 (55)	24 (44)	17 (46)	
Data missing	1 (2)	3 (5)	1 (3)	
Treatment stage IV				0.195[3]
ICI	31 (70)	40 (73)	32 (86)	
No ICI	13 (30)	15 (27)	5 (14)	
Stage IV treatment pre-PD-1i[2]				0.360[3]
No	25 (57)	29 (53)	25 (68)	
Yes	19 (43)	26 (47)	12 (32)	
Freckles				0.146[9]
No	34 (77)	38 (70)	20 (54)	
Yes	8 (18)	14 (25)	13 (35)	
Unanswered	2 (5)	3 (5)	4 (11)	
Hair colour				0.697[10]
Black/brown	9 (20)	10 (18)	8 (22)	
Blond	32 (73)	40 (73)	24 (65)	
Red	2 (5)	5 (9)	5 (14)	
Unanswered	1 (2)	0	0	
Tanning ability				0.630[11]
Dark/moderate	19 (43)	18 (33)	15 (41)	
Light/no tan	25 (57)	35 (64)	22 (60)	
Unanswered	0	2 (4)	0	
Times sunbathing each year				0.315[12]
< 15 times	34 (77)	47 (85)	30 (81)	
≥ 15 times	10 (23)	6 (11)	7 (19)	
Unanswered	0	2 (4)	0	
Level of education				0.715[3]
Upper secondary school or below	18 (41)	24 (44)	13 (35)	
Above upper secondary school	26 (59)	31 (56)	24 (65)	

SD, standard deviation; IQR, interquartile range; ICI, immune checkpoint inhibitor; PD-1i, programmed cell death 1 inhibitor; staging according to AJCC 8th edition.

1) Indicates site of distant metastasis according to AJCC 8th edition.

2) Indicates patients who initiated first-line treatment for stage IV disease or who should have initiated treatment prior to the introduction of PD-1i as standard treatment at the department.

3) Chi[2] test

4) ANOVA

5) Head/Neck vs trunk vs extremity, chi^2 test
6) Kruskal Wallis test
7) M1a + M1b vs M1c + M1d, chi^2 test
8) BRAF mutation vs wildtype, chi^2 test
9) Freckles vs no freckles, chi^2 test
10) Black/brown vs blond vs red, chi^2 test
11) Dark/moderate vs light/no tan, chi^2 test
12) < 15 times vs ≥ 15 times, chi^2 test

REFERENCES

1. Gandini S, Sera F, Cattaruzza MS, Pasquini P, Zanetti R, Masini C, et al. Meta-analysis of risk factors for cutaneous melanoma: III. Family history, actinic damage and phenotypic factors. Eur J Cancer 2005; 41: 2040-2059.
